# Erythropoietin in Optic Neuropathies: Current Future Strategies for Optic Nerve Protection and Repair

**DOI:** 10.3390/ijms23137143

**Published:** 2022-06-27

**Authors:** Yi-Fen Lai, Ting-Yi Lin, Pin-Kuan Ho, Yi-Hao Chen, Yu-Chuan Huang, Da-Wen Lu

**Affiliations:** 1 Department of Ophthalmology, Tri-Service General Hospital, National Defense Medical Center, Taipei 11490, Taiwan; iris92929@gmail.com (Y.-F.L.); pa735210@gmail.com (T.-Y.L.); doc30879@mail.ndmctsgh.edu.tw (Y.-H.C.); 2 School of Dentistry, National Defense Medical Center, Taipei 11490, Taiwan; james.sonicho@gmail.com; 3 School of Pharmacy, National Defense Medical Center, Taipei 11490, Taiwan; 4 Department of Research and Development, National Defense Medical Center, Taipei 11490, Taiwan

**Keywords:** erythropoietin, neuroprotection, retinal ganglion cell, optic neuropathy, optic nerve protection

## Abstract

Erythropoietin (EPO) is known as a hormone for erythropoiesis in response to anemia and hypoxia. However, the effect of EPO is not only limited to hematopoietic tissue. Several studies have highlighted the neuroprotective function of EPO in extra-hematopoietic tissues, especially the retina. EPO could interact with its heterodimer receptor (EPOR/βcR) to exert its anti-apoptosis, anti-inflammation and anti-oxidation effects in preventing retinal ganglion cells death through different intracellular signaling pathways. In this review, we summarized the available pre-clinical studies of EPO in treating glaucomatous optic neuropathy, optic neuritis, non-arteritic anterior ischemic optic neuropathy and traumatic optic neuropathy. In addition, we explore the future strategies of EPO for optic nerve protection and repair, including advances in EPO derivates, and EPO deliveries. These strategies will lead to a new chapter in the treatment of optic neuropathy.

## 1. Introduction

Erythropoietin (EPO) is a hormone that can stimulate erythropoiesis [1]. Its expression is regulated by hypoxia-inducible factors (HIF), a transduction factor sensitive to anemia and hypoxia [2]. EPO is produced by interstitial cells in the adult kidney [3]. It is secreted into the plasma and stimulates hematopoietic stem cell differentiation into red blood cells; however, the effect of EPO is not limited to erythroid tissues. EPO and EPO receptors (EPOR) have autocrine and paracrine functions in extra-hematopoietic tissues such as the endothelium, the heart, and the central nervous system, including the retina [4,5,6,7]. The role of EPO in paracrine signaling in the retina, which occurs inside the blood-retinal barrier, suggests its physiological roles other than erythropoiesis.

An abundance of EPO and EPOR has been demonstrated in the retina of humans [8]. EPO, the product of ganglion cells and retinal pigment epithelium cells is capable of targeting EPOR on photoreceptor cells, bipolar cells and amacrine cells [9]. A study indicated that EPOR upregulation is important for neuroprotection in retinal ischemic preconditioning [10]. Another study supported that exogenous EPO could protect neuron from damage in a model of transient global retinal ischemia [11]. In our previous studies, we found that EPO could protect cultured adult rat retinal ganglion cells (RGCs) against N-methyl-d-aspartate (NMDA) toxicity, tumor necrosis factor-alpha (TNF-α) toxicity and trophic factor withdrawal (TFW) [12]. Our in vivo study also found that intravitreal injection of EPO could attenuate NMDA-mediated excitotoxic retinal damage [13]. Except for the studies mentioned, many researchers have stated that EPO possesses antiapoptosis [14], antioxidative [15] and anti-inflammatory [16] properties. These properties are factors to why EPO is characterized to have neuroprotective effect in an organism’s retina. Understanding the features of EPO might result in the development of beneficial optic neuropathy treatments, including new delivery systems and derivatives with prolonged drug action of tissue protection and without erythropoietic side effects. Therefore, we summarize the available studies involving the neuroprotective effects of EPO in optic neuropathies and propose future strategies involving EPO in optic nerve repair and protection.

## 2. EPOR: Different Isoforms with Pleiotropic Functions

The structure of EPOR consists of a cytoplasmic domain with 235 amino acids, a single transmembrane domain with 23 amino acids, and an extracellular domain with 225 amino acids [17]. There are two subdomains, D1 and D2, in the extracellular domain, both of which are necessary for EPO binding [17]. Different isoforms of EPOR have been identified and characterized to have pleiotropic functions:

### 2.1. The Homodimer Isoform: EPOR_2_

**Figure 1:**
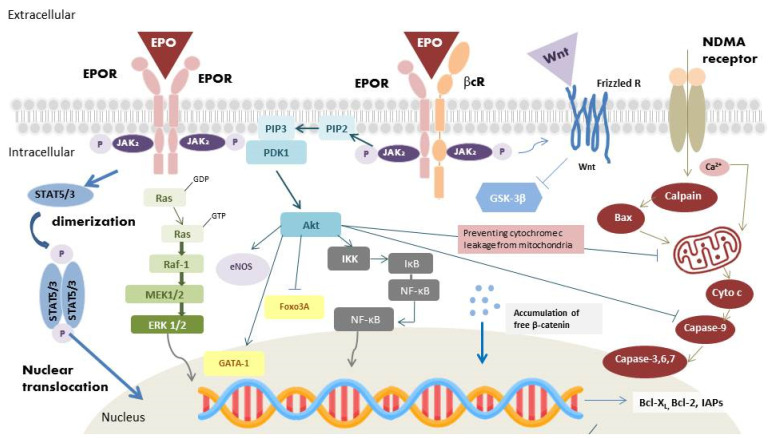
Binding of EPO to EPOR induces JAK-2 phosphorylation, dimerization, and subsequently activate STAT5/3, Ras/Raf/MEK/ERK, PI3-K/Akt, and NF-κB pathways. JAK-2 phosphorylates STAT5 or STAT3, leading to the dimerization of STAT5 (STAT3). STAT5 (STAT3) and the last signaling molecule in the MAPK pathway translocate into the nucleus and upregulate the expression of antiapoptotic Bcl-2 and Bcl-xL. Activation of PI3-k/Akt pathway increases endothelial nitric oxide synthase (eNOS) protein expression and NO production, which could increase blood flow and attenuate regional injury. PI3-k/Akt pathway also phosphorylates transcription factor GATA-1 and Foxo3 A, which enhance the expression of antiapoptosis proteins. Activation of the IKK complex by Akt phosphorylates IκB, resulting in its ubiquitination, and degradation, and in the releases of bound NF-κB. Free NF-κB translocates into the nucleus and exerts its antiapoptosis activity through the expression of inhibitors of apoptotic proteins (IAPs). Furthermore, binding of EPO to EPOR/βcR activates Wnt signaling, which inhibits GSK-3β phosphorylation and allow β-catenin to stabilize and accumulate in the cytoplasm in a non-phosphorylated form. Free β-catenin translocates into the nucleus and trigger transcription of Wnt-target gene responsible for cell antiapoptosis and the development of nervous system. Activation of NMDA receptors allows the influx of Ca^2+^, which induces excitotoxicity via initiation of the μ-calpain/Bax/cytochrome c/caspase-9 pathway. The caspases result in DNA fragmentation and lead to cell apoptosis. Activation of PI3-K/Akt pathway could also inhibit caspase activity by preventing cytochrome c leakage from mitochondria, thus inhibiting DNA degradation.

The homodimer isoform is present in erythroblasts [17]. During hematopoiesis, EPO binds to its receptor and results in homodimerization of EPOR (Figure 1) [18]. Following binding, Janus kinase-2 (JAK-2) activates several secondary signal molecules [17,19], such as STAT5 [20], MAPK, and PI3-K/Akt [21]. The activation of these molecules contributes to the differentiation and maturation of erythroid progenitor cells [22].

### 2.2. Heterodimer Isoform: EPOR/βcR

In addition to the homodimerization, there are other types of EPO receptors, which involve affinities 8–16 times weaker [23,24]. A study confirmed the receptor was a heterodimer consisting of EPOR and the β common receptor (βcR), a subunit of granulocyte- macrophage colony stimulating factor, interleukin 5, and interleukin 3 [25]. Since the EpoR/βcR heterodimer has the properties against tissue injury and inflammation, some authors named it as the “tissue-protective receptor” or “innate repair receptor” [26]. Recent data found the presence of this heterodimer complex in the RGCs, inner nuclear layer and photoreceptors [27]. In experimental studies, EPO binding to the EPOR/βcR heterodimer could reduce light-induced photoreceptor cell death [27]. Additionally, EPO binding to the EPOR/βcR heterodimer could activate Wingless (Wnt) signaling (Figure 1) [28], which could regulate cells survival, and differentiation. Wnt signaling is an important pathway responsible for the development of different ocular structure [29]. This heterodimer receptor contributes to the majority of the protective effects of EPO, which potentially underlines a vast quantity of therapeutic approaches.

### 2.3. Extracellular Soluble Isoform: sEPOR

The extracellular soluble isoform of EPOR (sEPOR), which lacks the transmembrane and cytoplasmic domains, is found in human plasma [30]. During hypoxia, in contrast to the expression of the full-length form increased through HIF transduction, the expression of the sEPOR is downregulated. In many studies, sEPOR is viewed as an endogenous antagonist of EPO, which blocks the neuroprotective effects of EPO. This form interacts with EPO without further activation of any downstream pathways. Moreover, its binding with EPO restricts the interaction of EPO with other receptor isoforms, resulting in a lower availability and bioactivity of EPO [10,31].

## 3. Effects of EPO

### 3.1. Angiogenic Effects

The transcription factor, HIF, has a vital role in hematopoiesis. In normoxic conditions, prolyl hydroxylase domain proteins (PHDs) hydroxylate all HIF-α subunit. After binding to von Hippel–Lindau tumor suppressor protein (VHL), hydroxylated HIF-α is then ubiquitinated. The ubiquitination of hydroxylated HIFs results in its degradation by the proteasome. In hypoxic conditions, the action of PHDs is inhibited. The stabilized HIF-α thus binds to HIF-β and translocates into the nucleus to regulate erythropoiesis via regulation of the expression of the EPO gene, vascular endothelial growth factor (VEGF) [32], as well as genes coding for proteins involved in iron metabolism [33], which are important for tissue oxygenation. After translation of EPO, the EPO is secreted into the circulation to reach hematopoietic cells. EPO binds with EPOR on erythroid cells, which triggers homodimerization of EPOR and activates the EPOR-associated JAK-2 by autophosphorylation (Figure 1) [17]. The active kinase JAK-2 results in the phosphorylation of tyrosine residues on the cytoplasmic portion of the EPOR [17]. The phospho-tyrosine residues recruit various proteins, which subsequently activate a series of pathways, including JAK-2/STAT5 (STAT3), PI3-K/Akt, and MAPK pathway [20,21]. JAK-2 phosphorylates STAT5 or STAT3, once it binds to the cytoplasmic portion of EPOR. STAT5 (STAT3) homodimerizes and translocates into the nucleus as a gene transcription factor. Activating the JAK-2/STAT5 (STAT3) pathway also leads to the upregulation of the antiapoptotic B-cell lymphoma-extra-large (Bcl-X_L_) protein, therefore protecting proerythroblasts from apoptosis [34]. After the activation of Ras by adaptor proteins, initiation of RAF/mitogen-activated protein kinase (MEK)/extracellular signal-regulated kinase (ERK) pathway would occur. RAF-1 protein kinase phosphorylates MEK, which subsequently phosphorylates MAPK/ERK1/2 [35]. The last molecules in the cascades translocate into the nucleus and activate various gene transcription factors for erythropoiesis regulation. PI3-K/Akt pathway is one of the main activating signaling pathways. PI3-K could lead to the phosphorylation of Akt, which could activate other proteins involved in erythropoiesis regulation. Akt phosphorylates the transcription factor GATA binding protein-1 (GATA-1), which is an important transcription factor for the anti-apoptotic Bcl-X_L_ expression and erythroid-specific genes. Phosphorylation of GATA-1 could enhance GATA-1 activity in erythroid cell [36]. The forkhead box O3A (Foxo3A), another Akt targeted transcription factor, has proapoptotic functions; in contrast, phosphorylation of Foxo3A inhibits its transcriptional activity [37]. Figure 1 illustrates the intracellular signaling pathway of EPO. Dysfunction in these signaling pathway leads to abnormal erythropoiesis by disrupting cells proliferation and apoptosis.

### 3.2. Antiapoptotic Effects

Binding of EPO to EPOR results in JAK2 phosphorylation and initiates STAT5 (STAT3), MAPK, PI3-K/Akt and nuclear factor kappa-light-chain-enhancer (NF-κB) downstream pathways, which execute the antiapoptotic effect of EPO (Figure 1) [38]. The last molecules in the STAT5 (STAT3) and MAPK pathways could translocate into the nucleus and activate the apoptotic regulators of Bcl-2 family, antiapoptotic Bcl-2 and Bcl-X_L_, to inhibit apoptosis [39]. Activation of PI3-K/Akt pathway also prevents cell apoptosis. Cell death signaling can be initiated by caspases or mitochondrial membrane depolarization. When the mitochondria membrane is depolarized, cytochrome c would be released into the cytoplasm and form the apoptosome complex with apoptotic protease activating factor-1 (Apaf-1) [40]. Pro-caspase-9 is activated by the apoptosome, which initiates downstream caspase activation. The activated caspases would cause DNA fragmentation and lead to cell apoptosis [40]. Activation of PI3-K/Akt pathway could inhibit caspase activity by preventing cytochrome c leakage from the mitochondria [41]. IκB kinase (IKK), another Akt target, is also associated with cell survival. In resting cells, NF-κB is held by the IκB. Activation of the IKK complex phosphorylates IκB, resulting in its ubiquitination and degradation, and in the releases of bound NF-κB. NF-κB exerts its protective effects through the increase in inhibitors of apoptotic protein (IAPs) [42], blocking of caspase activity [42], suppression of TNF-α related apoptosis [42], direct enhancing activation of Bcl-X_L__,_ and removal of cellular reactive oxygen species (ROS) [43].

In addition, Wnt signaling was proven to inhibit cancer therapy-mediated apoptosis and exhibit its oncogenic properties through the antiapoptosis effect [44]. Binding of EPO to EPOR/βcR heterodimer, present in RGCs and ocular stem cells, also activates Wnt signaling (Figure 1). Wnt binds to the Frizzled transmembrane receptors, which inhibits β-catenin phosphorylated by glycogen synthase kinase (GSK)-3β [45]. Free β-catenin accumulates and thus translocate into the nucleus. The binding of β-catenin to T-cell factor (Tcf) regulates cells survivability [44].

### 3.3. Anti-Inflammatory Effects

In inflammatory conditions, EPO was detected at the borders of the injury sites. Hence, the potential anti-inflammatory effect of EPO has also been investigated. EPO was found to decrease pro-inflammatory cytokine production, including intercellular adhesion molecule-1 (ICAM-1) [46], interleukin-6 (IL-6) [47], and TNF-α [48,49]. EPO also increased the production of the anti-inflammatory cytokine IL-10 [48]. Additionally, EPO could increase endothelial nitric oxide synthase (eNOS) protein expression (Figure 1) [50], which increase nitric oxide production. Nitric oxide could increase blood flow, and attenuate regional injury [51]. As regards the innate immune system, EPO could facilitate phagocytosis in macrophages [52], mediate dendritic cell maturation and immunomodulation [53], and reduce inflammation caused by mast cells [54]. In previous literature, these effects were thought to be mediated by the inhibition of pro-inflammatory cytokines. However, recent studies have confirmed the existence of EPORs on human T and B lymphocytes, suggesting that EPO could potentially have a direct impact on the immune cells [55]. In the adaptive immune system, EPO could directly promote the proliferation of regulatory T cells, but inhibit the proliferation of conventional T cells without inducing apoptosis [56]. These anti-inflammatory effects of EPO have been observed in several experimental studies of kidney transplant, colitis and encephalomyelitis [56,57,58]. Thus, EPO is thought to be an important hormone that facilitates immune homeostasis.

### 3.4. Antioxidant Effects

EPO has the ability to attenuate oxidative stress, allowing it to be categorized as a cytoprotective agent [59,60]. EPO could induce heme oxygenase-1 expression via PI3K/Akt pathway [61], which could provide a cytoprotective effect in astrocytes [62]. EPO also increases the level of glutathione peroxidase, a potent antioxidant protein, which can decrease the toxic activity of ROS [63]. Apart from direct antioxidative effects of EPO, indirect antioxidative effects have been reported. For example, the increase in the number of red blood cells resulting from EPO activity results in an increase in total level of antioxidative enzymes. [64]. EPO could also indirectly inhibit iron-dependent oxidative injury by depleting iron, a major catalyst for free radial reaction [65].

## 4. Current Strategy of EPO for Optic Nerve Protection and Repair

Encouraging results of EPO from basic research support the possibility of integrating its therapeutic effects in glaucomatous optic neuropathy, optic neuritis, non-arteritic anterior ischemic optic neuropathy (NAION), and traumatic optic neuropathy (TON). We summarize the available studies in the literature on the use of erythropoietin in these optic neuropathies (listed in Table 1).

**Glaucomatous optic neuropathy**, a neurodegenerative disease, is characterized by progressive loss of RGCs. Elevated intraocular pressure (IOP) is considered the most important risk factor of glaucomatous optic neuropathy. However, some patients experienced continued RGCs loss despite good intraocular pressure control, suggesting the presence of other complicated mechanisms stimulating RGC death. Multifactorial mechanisms have been postulated for glaucomatous optic neuropathy, including vascular insufficiency, inflammation [66,67], excitotoxicity [68] and neurotrophic factor withdrawal [69]. Due to the complex pathogenesis of glaucoma, EPO was developed to prevent the IOP-independent RGCs loss. Several studies have reported that the EPO level in the aqueous humor increased in patients with glaucoma [70]. The cause of the elevated aqueous EPO in glaucomatous eyes might be related to the ischemia, hypoxia, or elevated ROS caused by glaucomatous damage [71]. The increase in EPO is identified as a compensatory response due to the presence of glutamate, nitric oxide and the free radicals after the glaucomatous damage [72].

Previous studies have reported glutamate and NMDA excitotoxicity as the probable mechanism of glaucoma. This involves the opening of ion channels which allows the entry of extracellular Ca^2+^ into neurons. Ca^2+^ acts as second messenger to activate downstream signaling pathways leading to RGCs apoptosis [73]. TNF-α and TNF-α receptor 1 signaling could also induce RGC hyper-excitability by upregulating Na^+^ channels, which contribute to RGCs apoptosis in glaucoma [74]. In our previous study, we cultured RGCs from adult rats in a medium containing neurotrophic factors [12]. Cytotoxicity was induced by NMDA, TFW, and TNF-α. EPO was found to provide neuroprotection to cultured adult rat RGCs against NMDA-, TFW-, and TNF-α -induced toxicity. The efficacy of EPO is similar with memantine (an NMDA receptor antagonist), glial cell-derived neurotrophic factor (GDNF), and Z-IETD-FMK (a caspase-8 inhibitor). Additionally, inhibiting STAT5, MAPK/ERK and PI3K/Akt signal impaired the protective effects of EPO [12]. We subsequently investigated the effect of EPO in vivo study [13]. Wistar rats were randomly assigned to different groups treated with intravitreal NMDA and EPO. We found that EPO had dose-dependent neuroprotective effect against NDMA-mediated neurotoxicity. Through histological findings, EPO was also found to reverse the NMDA-induced damage to bipolar cell axon terminals in the inner plexiform layer. We also observed that in the excitotoxic signaling pathway of NDMA-induced toxicity, μ-calpain is activated first, followed by Bax, and then caspase-9 (Figure 1). EPO could protect RGCs by downregulating the activity of μ-calpain, Bax as well as caspase-9 [13].

Apart from our previous research results, EPO was also found to be neuroprotective via systemic, intravitreal, subconjunctival and retrobulbar administration in rat model of glaucoma. The DBA/2J mice, which spontaneously develop glaucomatous loss of RGC and are used to mimic human hereditary glaucoma, were intraperitoneally injected with EPO. Treatment with EPO could promote RGC survival without affecting IOP [75]. Subconjunctival injection of EPO in a rat model of glaucoma demonstrated increase in electroretinography wave amplitudes and retinal thickness [76]. Retrobulbar injection of EPO could preserve RGCs in rats with acute elevated IOP [77]. A single intravitreal injection of EPO could provide protective effects on RGC viability in rat model of glaucoma [78]. Based on the aforementioned studies, EPO is found to have neuroprotective effects regardless of the EPO administration methods. However, the discussion of EPO in the treatment of glaucoma is limited to animal studies. In humans, there are only a few observational studies investigating the correlation between EPO and glaucoma, especially neovascular glaucoma [79,80,81]. To date, human studies using EPO for the treatment of glaucoma are still lacking. Future studies could focus the application of EPO in patients with primary open-angle glaucoma to see if EPO exhibits the same neuroprotective effects in animal experiments.

**Optic neuritis** is another high occurring disease among the world population. For optic neuritis, methylprednisolone is the standard treatment in clinical practices. Although steroid treatment could accelerate visual acuity recovery, recent study demonstrates that steroids could not influence the visual outcome or atrophy of the optic nerve [82]. An animal study even demonstrated that methylprednisolone could increase RGCs degeneration by inhibiting the neurotrophin pathway [83]. Since EPO has shown multiple neurotrophin-like properties in various neuronal disorders, the efficacy of EPO is evaluated as an add-on therapy to methylprednisolone in autoimmune optic neuritis by investigators. In an experimental autoimmune encephalomyelitis (EAE) rat model, intraperitoneal injection of EPO (5000 U/kg) significantly increased the survivability and functionality of RGCs in rats afflicted with myelin oligodendrocyte glycoprotein (MOG)-induced optic neuritis [84]. In the model of MOG-EAE, Sättler et al. concluded that the PI3-K/Akt pathway plays an important role in RGCs survivability under systemic treatment with EPO [84]. Establishment of potentially relevant intracellular conduction pathways might make the application of EPO more feasible in MOG-EAE. Human studies have been performed, but the results were not conclusive. A comparatives study in humans demonstrated no difference in visual acuity, visual field and contrast sensitivity between the intravenous EPO (20,000 IU/day) accompanied with methylprednisolone group, and the methylprednisolone only group [85]. A comparative study reported intravenous EPO (33,000 IU/day) as an add-on therapy to methylprednisolone improved median deviation of perimetry in acute optic neuritis [86], but post-intervention retinal nerve fiber layer (RNFL) thickness demonstrated no significant difference from the methylprednisolone only group [86]. One double-blinded randomized control study demonstrated decreased structural and functional impairments in EPO add-on group [87]. Retinal nerve fiber thinning was less apparent, and visual evoked potential latencies were shorter in the EPO add-on group than in the control group. One randomized, placebo-controlled, double blind, phase 3 study compared patients receiving intravenous EPO (33,000 IU/day) plus methylprednisolone to patients receiving placebo plus methylprednisolone [88]. Mean RNFL thickness atrophy and mean low contrast letter acuity scores showed no difference between these two groups [88]. Most of the studies failed to demonstrate EPO to be a structurally and functionally neuroprotective agent as an add-on therapy in optic neuritis. The reason might be that most of these studies chose longer disease-treatment duration (0–10 days), and did not stratify the severity of optic neuritis. The application of EPO on the injured tissues might lack receptors activity since severe inflammation might decrease tissue bioavailability for drugs to interact. Additionally, all studies administrated EPO systemically in a short duration (3 days). The efficacy of EPO might therefore be limited. Future studies into this matter could classify the severity of optic neuritis, administer EPO more closely to disease onset and extend the treatment duration to see the therapeutic effects of EPO.

**Non-arteritic anterior ischemic optic neuropathy** is thought to result from vascular insufficiency. Patients with hypertension or obstructive sleep apnea have higher risk of developing NAION since the disease could result in hypoperfusion of the optic nerve. The hypoperfusion causes ischemia and swelling of the axons, thus increasing the pressure on the nervous tissues confined within the tight borders of the posterior scleral outlet. The axon swelling results in further ischemia and neuron swelling. The vicious cycle leads to severe ganglion cells damage. Due to the evidence showing neuroprotection effect of EPO, investigators also determined the efficacy of EPO in NAION. Modarres et al. conducted a prospective interventional case series by intravitreally injecting EPO (2000 IU/0.2 mL) into thirty-one patients within 1 month of the NAION onset [89]. Within the first month, 61.2% of patients had shown improvement in visual acuity, after 3 months, the protective effect of EPO began to wear off. Nevertheless, the visual acuity remained significantly better than baseline after a 6-month follow up [89]. Pakravan et al. performed another prospective comparative case series in 113 patients diagnosed as recent onset NAION (less than 14 days) [90]. Patients were categorized into three groups: intravenous methylprednisolone with intravenous EPO (10,000 IU twice a day for 3 days), intravenous methylprednisolone, and control group. Among the three experimental groups, there were no statistically significant differences in best-corrected visual acuity (BCVA), mean deviation, and peripapillary RNFL thickness after a 6-month follow up. The same research group later performed a randomized clinical trial to compare the effect of systemic EPO (10,000 IU twice a day for 3 days) versus oral steroids (75 mg daily tapered off within 6 weeks) versus placebo [91]. A total of 99 patients diagnosed as acute-onset (<5 days) NAION were included. The EPO-treated group did not improve visual acuity and mean deviation of visual field when compared to the oral steroid-treated group and placebo group. However, more patients (55%) in the EPO group gained at least three lines of BCVA. Patients in EPO group preserved more peripapillary RNFL [91]. Among the aforementioned studies, the case series by Modarres et al. reported that EPO was beneficial in NAION, but its limitation was the lack of a comparison group. The subsequent interventional comparative study by Pakravan et al. failed to demonstrate the benefits of EPO in NAION. They were debated involving the concomitant use of systemic steroid and EPO because high-dose steroid has shown to inhibit pro-inflammatory cytokines and neurotrophic factors. The postulated systemic steroid might blunt EPO’s neuroprotective effects. Limitations of the study include the lack of randomized study design and the broad inclusion window (14 days), so the neuroprotective effect of EPO might not be demonstrated. The research team subsequently improved the limitations of their study by publishing a randomized study and narrowing the inclusion window (5 days), which proved that EPO did have some structural and functional benefits although EPO group did not have significantly better visual acuity than that in the steroid and placebo groups at the end of the tracking. Since existing studies shows intravenous EPO appears to have limitations in the treatment of NAION, future studies should be directed toward a larger randomized study to replicate the benefit of intravitreal EPO in NAION in Modarres’s study.

For **traumatic optic neuropathy**, indirect TON is the more common type. The shearing force could lead to small vessel and neuron axon injury around the optic nerve by inducing ischemia, inflammation, and oxidative stress, all of which result in ganglion cell death. Currently, the common treatments are observation, corticosteroids and optic canal decompression. However, none of these managements are proven to be effective. Since EPO has shown to be neuroprotective, EPO might play a role in treating indirect TON. Intravenous EPO was first commenced in patients with indirect TON by Kashkouli et al. in 2011 [92]. Indirect TON patients with intravenous EPO (10,000 IU in 3 days) were compared to indirect TON patients without treatment. They found that the EPO-treated group has higher BCVA than that in the observation group. They advocated intravenous EPO may be a new effective and safe treatment in patients with indirect TON [92]. The efficacy of EPO in indirect TON was re-tested by Enterzari et al. in 2014. In the case series, EPO was also shown to improve the mean BCVA [93]. In 2017, Kashkouli et al. performed a phase 3, multicenter study. They enrolled TON patients with trauma-treatment duration less than 3 weeks [94]. The mean BCVA was compared among the three groups, including the EPO group, the methylprednisolone group and the observational group. The dosage of EPO was given according to patient’s age, where 10,000 units EPO per day were infused into patients under 13 years of age and 20,000 units EPO per day were infused into patients above 13 years of age for 3 consecutive days. The EPO-treated group has better color vision than other groups. All three groups demonstrated improvement of BCVA. Although a better final vision was seen in the EPO group, but the results were insignificant between the groups [94]. They also reported late treatment (>3 days) and initial BCVA of no light perception as poor prognosis factors, but then another study takes a different view. Another study by Rashad et al. investigated the efficacy of intravitreal EPO in treating recent (<3 months trauma-treatment duration) and old (3–36 months) TON in 2018 [95]. They reported intravitreal injection of EPO (2000 IU/0.2 mL) improved BCVA, visual evoked response amplitude and latency in either recent and old indirect TON [95]. All of the above studies reported EPO could improve BCVA in TON patients either in the case series or in a larger clinical trial. Although EPO seems to bring promising experiment results, these studies still lack randomized study designs. The results need to be interpreted with care. The American Academy of Ophthalmology presented a report exploring the efficacy of surgery, steroids, EPO and other drugs for TON; however, they were also unable to reach a conclusion due to lack of level I evidence [96]. Notably, Rashad’s study reported that intravitreal EPO could improve vision in old TON patients. The conclusion is highly anticipated since there has been no effective treatment for old TON patients. Future studies should prioritize a large, randomized study, while investigating the efficacy of intravitreal EPO in recent or old TON patients.

## 5. Advances in EPO Derivatives

For more than a decade, the use of EPO to treat hematopoietic anemia in chronic kidney disease has played an integral role in clinical practice. On non-hematopoietic cells, high-dose systemic EPO administration is required to promote tissue repair and neuroprotection due to the low affinity toward heterodimeric EPOR/βcR [23]. However, high doses of EPO have the potential to trigger undesirable side effects such as polycythemia and thromboembolic events. The development of EPO derivatives with a higher affinity toward the heterodimeric EPOR/βcR would further improve medical protocols by eliminating undesirable and detrimental effects. In addition, the availability of EPO derivatives could potentially lower the costs of EPO treatment and provide new series of treatment options to counteract different neurodegenerative diseases. Recently, newly modified EPO possesses improved characteristics as an erythropoiesis-stimulating agent (ESA), including diminished side effects, extended half-life, and reduced clearance rate during circulation.

**Epoetin alfa** (Epogen), a type of ESA medicine, has been the standard of care for patients with kidney disease and cancer-related anemia. **Epoetin alfa-epbx** (RetacritTM) shares the same amino acid sequence and similar carbohydrate composition as epoetin alfa (EpogenTM). In 2018, the protein was approved by the FDA, making it the first biosimilar EPO molecules approved in the USA [97]. **Darbepoetin alfa** (DA, Aranesp), an alternative agent of Epoetin alfa and a hyperglycosylated EPO analog, is a novel ESA with two additional N-glycosylation sites accompanied by 22 sialic acid moieties. In the attempt to extend the molecule’s half-life by three-fold longer than EPO in vivo, glycoengineering was conducted to increase the structure’s resistance to degradation. Darbepoetin alfa was approved for treating anemia resulting from renal diseases and cancer chemotherapy. The treatment protocol only requires a once-per-week visit and is accompanied by lower clinical costs [98,99]. **C.E.R.A.** (continuous erythropoietin receptor activator), a third-generation ESA, is an EPO (~34 kDa) integrated with methoxypolyethylene glycol (PEG, 30 kDa). Compared with other EPO derivatives, C.E.R.A. has a unique pharmacological profile with the longest half-life and slowest clearance rate. These unique pharmacological properties exist because of methoxypolyethylene glycol (PEG) integration into EPO. Notably, EPO pegylation (the process of connecting a hydrophilic polymer to EPO) significantly prolongs the duration of EPO action, and enhances proteolytic resistance in cell-free plasma [100].

Two types of modified EPO molecules with no affinity towards canonical EPOR have been developed, each of which possesses tissue protective effects by binding onto heterodimeric EPOR/βcR. The two enzymatically desialyated EPO are asialoerythropoietin (asialoEpo) and carbamylated EPO (cEpo), with each having neuron and oligodendrocyte protection capabilities without erythropoietic functions. **Asialerythropoietin** (asialoEPO) was evaluated to be a safe drug for clinical treatments. However, asialoEPO’s half-life (t1/2~1.14 min) is much shorter than that of EPO (t1/2~5.6 h). The short half-life gives asialoEPO insufficient persistence time to stimulate hematopoiesis. Based on the above concept, researchers found that chemical modification of the EPO binding sites could abolish erythropoiesis function but retain the tissue-protective effect. **Carbamylated EPO (cEpo)**, a chemically modified derivative of EPO’s lysine residues, was found to act through the heterodimeric EPOR/βcR rather than classical EPOR_2_ primarily because of the modified structure of cEpo. The study has confirmed that cEpo possesses neuron anti-apoptotic effects similar to EPO but instead does not induce neovascularization [101]. Investigators emphasized the future pharmacological role of cEpo as a non-hematopoietic neuroprotective agent. In recent years, the neuroprotective effects of cEpo makes it a rising candidate for prospective drugs [102].

Helix B of EPO, exposed to aqueous medium away from the binding sites of EPO and EPOR2, is important for the recognition of heterodimer EPOR/βcR. Based on the finding, investigators developed an eleven-amino acid linear peptide, mimicking the structure of the external surface of the helix B peptide and named it as ARA290 or Cibinetide or **helix B surface peptide (HBSP)**. As predicted, HSBP were not erythropoieitic but has properties in protecting against neuronal injury. McVicar et al. demonstrated that HBSP is sufficient in activating tissue-protective pathways without altering hematocrit or exacerbating neovascularization [103]. Although it has clear advantages, the 2 min plasma half-life of HBSP limits its application in vivo. Based on the amino acid sequence of HBSP, Zhang et al. designed and synthesized **thioether-cyclized helix B peptide (CHBP)** to increase structure integrity, prevent proteolytic degradation, and improve tissue-protective potency [98,104]. More recently, Cho et al. further designed a next-generation modified helix C peptide (**ML1-h3**) capable of improving neuroprotective effects against oxidative stress. This innovation would promote EPOR-mediated cell survival and proliferation in vitro and in vivo. This process signifies a brighter prospective for clinical applications and promotes the value of developing EPO derivatives for clinical use [105].

## 6. Advances in EPO Delivery

### 6.1. Protein-Based Ocular Delivery

Many EPO studies involve frequent injections of ophthalmic proteins via invasive procedures, which might result in a variety of adverse effects and increase the probability of irreversible damage to the patient’s eye. Topical sustain released formulations are non-invasive drugs, that effectively reaches the posterior segment of the eye. According to Silva et al., mucoadhesive polymers such as chitosan and hyaluronic acid can improve the ocular bioavailability of drugs with the support of nanoparticulate delivery systems [106]. The formulation was found to be non-cytotoxic toward ARPE-19 and HaCa T cell lines. CS/HA6-rhEPO may be a promising topical formulation after enhancing its bioavailability through different ocular barriers. For the intraocular route of administration, De Julius et al. developed two polymer microparticles, poly (propylene sulfide) (PPS) and poly (lactic-co-glycolic acid) (PLGA), to prolong His-tagged rhEPO-R76E (42kDa) release [107]. The rhEPO-R76E was loaded into the polymeric microparticles to prolong in vivo release for at least 28 days to resolve the issue involving short half-life of the rhEPO-R76E (t1/2~13 min). PPS-based microparticles platform is especially promising because it is degradable by ROS. The delivery system provides extended neuroprotection and inherent antioxidant benefits, which reinforces its ability in ocular delivery of EPO.

### 6.2. Gene-Based Ocular Delivery

Under the gene delivery approaches, EPO has significant therapeutic potential in neurodegenerative diseases due to its neuroprotective effects. However, recombinant EPO is limited in clinical treatment of glaucoma patients due to its short half-life. As regards this issue, Bond et al. constructed a viral gene delivery system for EPO-R76E [108]. Treatment with recombinant adeno-associated virus (rAAV) provides sustainable, long-term delivery of EPO-R76E without a critical rise in hematocrit [108,109]. AAV-mediated long-term EPO expression is achievable in animal models with the primary functions of promoting red blood cells proliferation and neuroprotection.

Another challenge in applying gene therapy in humans is the improvement of drug selectivity. For systemically secreted hormone, such as EPO, it is vital to use an inducible genetic delivery system to avoid excess expression and side effects. However, precise expression control is highly desirable when maintaining steady-state red blood cell counts within a narrow therapeutic window. Hines-Beard et al. packaged EPOR76E into a recombinant adeno-associated viral vector under the control of the tetracycline inducible promoter [110]. In the retina, tetracycline-controlled expression of green fluoresce protein (GFP) in retinal pigmented epithelium and photoreceptor cells becomes apparent in rats following subretinal injections of rAAV-2/2 vector. The outer nuclear layer in the eyes was approximately 8 μm thicker in mice that received doxycycline water as compared to the control groups.

The morpholino-regulated hammerhead ribozyme was selected as another inducible genetic delivery system by Zhong et al. [111]. One of the designed ribozymes enabled regulation of AAV-delivered transgenes, allowing dose-dependent and more than 200 folds of protein to be expressed. The induction rate of morpholino becomes functional when interacting within EPO-encoding switchable AAV vectors. By controlling the dose of morpholino, EPO levels can be maintained for several weeks after a single injection while preventing hematocrit level fluctuations.

### 6.3. Surface Receptor-Targeted Ocular Delivery

Through surface receptor-targeted delivery results, most suggest that the tissue-protective effect of EPO and injury response are mediated by the EPOR/βcR heterodimer and not by the EPOR homodimer [112]. In addition to some well-known derivatives of EPO with better affinity for the EPOR/βcR heterodimer, such as asialoEPO, cEpo and HBSP (see above), traptamers of transmembrane domain (TMD) proteins of EPOR/βcR are also an option. He et al. constructed ELI-3 traptamer that specifically targets the TMD of human EPOR and triggers cooperative JAK/STAT signaling for proliferation and tissue protection [113]. Moreover, ELI-3 fails to induce erythroid differentiation from primary human hematopoietic progenitor cells. This traptamer-mediated delivery strategy not only provides selective receptor binding but also enhances binding affinity, and facilitates better EPO delivery efficiency.

### 6.4. Cell-Based Ocular Delivery

Mesenchymal stem cell (MSC) therapy is potential in treating optic neuropathies. MSCs have demonstrated to possess neuroprotective effects in numerous neurodegenerative diseases while maintaining retinal morphology [114,115]. They could also regulate inflammatory responses [116] and increase the secretion of neurotrophic factors [117]. Furthermore, they could transdifferentiate into retinal progenitor cells [118]. Johnson et al. intravitreally transplanted MSCs in a rat model of glaucoma and found an increased in axon survivability and decreased in axon loss of RGCs [119]. However, successful stem cell transplantation depends on the survivability of MSCs in a pathological environment. This can be addressed using EPO, which could enhance the engraftment of MSCs [120]. MSCs could be the vector of EPO because these cells could cross the brain-retinal barrier and localize into the inflamed sites [121]. The phenomenon emphasizes a mutualistic relationship between MSCs and EPO. Hence, some investigators attempt to evaluate the efficacy of EPO-expressing MSC in treating retinal degenerative diseases. Ding et al. transduced MSCs with lentiviral particles encoding EPO. They found that co-treatment with EPO and MSCs instead of only with MSC could attenuate human retinal neuron apoptosis by restoring the mitochondrial membrane potential and protect human retinal neurons from glutamate neurotoxicity [122]. The ability for EPO to synchronize with MSCs may become beneficial in producing medical protocols involving the treatment of patients with glaucomatous optic neuropathy. Given their endogenous long-term therapeutic effects, MSCs-based therapy could be the future direction in optic nerve repair.

## 7. Conclusions

EPO plays a vital role from the time of eye development to the many protective actions in optic neuropathies. EPO binding to heterodimeric EPOR/βcR initiates intracellular pathways, including JAK-2/STAT5 (STAT3), PI3-k/Akt, MAPK, NF-κB and Wnt signaling, which contributes to its anti-apoptosis, anti-inflammation, anti-oxidative effects. The above effects have brought promising neuroprotective results in several pre-clinical studies of glaucomatous optic neuropathy, optic neuritis, NAION and TON. However, the practical application of EPO is still limited by its hematopoietic side effects due to the lower affinity toward EPOR/βcR heterodimer. For this purpose, investigators developed EPO derivatives with extended half-life in plasma, decreased clearance rate and higher affinity toward EPOR/βcR heterodimer. These strategies enhance the tissue protection and reduce erythropoiesis. In addition, creating new EPO delivery systems is also a hurdle required to address. The development of topical drugs, microsphere, specific traptamer toward transmembrane domain of EPOR/βcR heterodimer, promoter-regulated gene therapy and even more appealing candidate, EPO-modified MSC therapy are promising therapeutic strategies in the future, which would bring endogenous long-term therapeutic effect in current noncurable optic degenerative diseases. We foresee that more clinical trials will be conducted to address on the safety and efficacy of these new strategies. Incorporating these therapeutic approaches with a better-controlled regulation could potentially magnify the beneficial effect of EPO for optic neuropathies in the future.

## Figures and Tables

**Table 1:** Summary of clinical studies that evaluate the effect of erythropoietin on optic neuropathies.

Authors	Year	Study Design	Number of Eyes/Patients (Animals)	Intervention	Main Outcomes
**Glaucoma (Animal Studies)**					
Cheng et al. [13]	2020	Randomized intervention study	125 Wistar rats	Randomly assigned into five groups: (1) Control (2) Intravitreal NMDA80 (3) Intravitreal NMDA80 + 10 ng EPO (4) Intravitreal NMDA80 + 50 ng EPO (5) Intravitreal NMDA80 + 250 ng EPO	EPO protects RGCs and bipolar cell axon terminals in IPL by downregulating apoptotic factors to attenuate NMDA-mediated excitotoxic retinal damage.
Zhong et al. [75]	2007	Intervention study	91 C57BL/6J mice and 294 DBA/2J mice	Assigned into 5 groups: (1) Control (2) Intraperitoneal Memantine (70 mg/kg/wk) (3) Intraperitoneal EPO (3000 IU/Kg/wk) (4) Intraperitoneal EPO (6000 IU/Kg/wk) (5) Intraperitoneal EPO (12,000 U/Kg/wk)	EPO’s effects were similar to those of memantine, a known neuroprotective agent. EPO promoted RGCs survival in DBA/2J glaucomatous mice without affecting IOP.
Resende et al. [76]	2018	Comparative study	26 Wistar Hannover albino rats with unilateral glaucoma induced by coagulation of 3 episcleral veins in the right eye Case (right eye): 13 eyes Control (left eye): 13 eyes	Subconjunctival injection of 1000 IU EPO versus placebo	EPO improved both scoptopic and photopic amplitude. Retinal thickness is thicker in EPO group.
Zhong et al. [77]	2008	Intervention study	75 rats with unilateral glaucoma induced by saline infused into anterior chamber. The IOP was raised to 70 mm Hg for a duration of up to 60 min.	Assigned into 5 groups: (1) Unoperated control (2) Operated control (3) Acute elevated IOP group (4) Acute elevated IOP + retrobulbar EPO (1000 U/100 μL) (5) Acute elevated IOP + vehicle solution retrobulbar injection (i.e., EPO diluted in a vehicle solution)	EPO with a retrobulbar administration could protect RGCs from acute elevated IOP.
Tsai et al. [78]	2005	Intervention study	29 Sprague Dawley rats with EVC glaucoma model	Assigned into 4 groups: (1) Unoperated control (2) Episcleral vessel cautery (3) EVC + intravitreal normal saline (4) EVC + intravitreal EPO(200 ng/5 μL)	RGC counts were significantly decreased in both the EVC and EVC+ intravitreal normal saline groups but not significantly decreased in the EVC-EPO treated retinas.
**Optic Neuritis (Human Studies)**					
Sanjari et al. [85]	2019	Nonrandomized comparative case–control study	62 patients with isolated retrobulbar optic neuritis (onset <10 days) Cases: 35 patients Control: 27 patients	Intravenous EPO 20,000 IU/day for 3 days + intravenous methylprednisolone versus intravenous methylprednisolone	No difference was observed between the two groups in BCVA, contrast sensitivity, MD of visual field, and pace of recovery of visual acuity at 120-day follow-up.
Shayegannejad et al. [86]	2015	Nonrandomized comparative case–control study	30 patients with acute optic neuritis with unknown origin or demyelinative origin (onset < 4 days) Cases: 15 patients Control: 15 patients	Intravenous EPO 33,000 IU/day for 3 days + intravenous methylprednisolone versus intravenous methylprednisolone	The amount of MD improvement was significantly higher in EPO-treated group. No difference was observed between the two groups in post-intervention PSD, amount of PSD improvement, post-intervention RNFL, and RNFL loss at 6-month follow up.
Sühs et al. [87]	2012	Randomized double-blind clinical trial	37 patients with unilateral optic neuritis (onset < 10 days) Case: 20 patients Control: 17 patients	Intravenous EPO 33,000 IU/day for 3 days + methylprednisolone versus intravenous methylprednisolone	EPO group had less RNFL thinning, shorter VEP latencies, and smaller decrease in retrobulbar diameter of optic nerve. No difference was observed between the two groups in recovery of visual acuity and visual field perception at 16-week follow-up.
Lagrèze et al. [88]	2021	Randomized double-blind clinical trial	103 patients with unilateral optic neuritis (onset < 10 days) Case: 52 patients Control: 51 patients	Intravenous EPO 33,000 IU/day for 3 days + methylprednisolone versus intravenous methylprednisolone	No difference was observed between the two groups in post-intervention RNFL thickness, low contrast visual acuity at 26-week follow up. One patient in EPO group developed a venous sinus thrombosis, which was treated with anticoagulants and resolved without sequelae.
**Non-Arteritic Anterior Ischemic Optic Neuropathy(Human Studies)**					
Modarres et al. [89]	2011	Case series	31 patients with NAION (onset ≤ 1 month)	Intravitreal injection of EPO (2000 IU/0.2 mL).	EPO improved visual acuity and MD at 3-month follow up. The effect of EPO began to wear off after 3 months. The improvement in BCVA from baseline persisted at 6-month follow-up.
Pakravan et al. [90]	2017	Nonrandomized comparative case series	113 patients with NAION (onset < 14 days) I.V. Steroid + EPO: 40 patients I.V. Steroid: 43 patients Observation: 30 patients	Assigned into 3 groups: (1) Intravenous EPO 10,000 IU BID for 3 days + intravenous methylprednisolone (2) Intravenous methylprednisolone (3) Observation	No significant differences were observed among the three groups in visual acuity, peripapillary RNFL thickness, and visual field at 6-month follow-up.
Nikkhan et al. [91]	2020	Randomized clinical trial	99 patients with NAION (onset ≤ 5 days) EPO: 34 patients Oral steroid: 33 patients Placebo: 32 patients	Assigned into 3 groups: (1) Intravenous EPO 10,000 IU BID for 3 days (2) Oral prednisolone (3) Placebo	More patients in the EPO group gained at least 3 lines of BCVA. Patients in EPO group preserved more peripapillary RNFL. No significant differences in visual acuity and MD of visual field among the three groups at 6-month follow-up.
**Traumatic Optic Neuropathy(Human Studies)**					
Kashkouli et al. [92]	2011	Nonrandomized comparative case–control study	15 patients with iTON (onset < 3 weeks) EPO: 7 patients Observation: 8 patients	Intravenous injection of EPO (10,000 IU/day) for 3 days	BCVA was significantly higher in the EPO group at last follow up (mean follow up time: EPO group: 7.0 months, observation group: 5.8 months)
Enterzari et al. [93]	2014	Case series	18 patients with iTON (onset < 2 weeks)	Intravenous injection of EPO (20,000 IU/day) for 3 days	EPO improve BCVA at 3-month follow-up.
Kashkouli et al. [94]	2017	Clinical trial	100 patients with TON (onset < 3 weeks) EPO: 69 patients Steroid: 15 patients Observation: 16 patients	Assigned into 3 groups: (1) Intravenous EPO (10,000 or 20,000 IU/day) for 3 days (2) Intravenous methylprednisolone (3) Observation	All three groups showed a significant improvement of BCVA. Differences between groups were not statistically significant. Color vision was significantly improved in the EPO group at 3-month follow-up.
Rashad et al. [95]	2018	Case series	Recent iTON (<3 month): 7 eyes Old iTON (3–36 months): 7 eyes	Intravitreal injection of EPO (2000 IU/0.2 mL)	Both groups have improvement in BCVA, visual evoked response amplitude, and latency at 6-month follow-up.

NMDA: N-Methyl-D-aspartic acid; EPO: erythropoietin; RGC: retinal ganglion cell; IOP: intraocular pressure; EVC: episcleral vessel cautery; BCVA: best-corrected visual acuity; MD: mean deviation; PSD: pattern standard deviation; RNFL: retinal nerve fiber layer; VEP: visual evoked potentials; NAION: non-arteritic anterior ischemic optic neuropathy; TON: traumatic optic neuropathy.

## Data Availability

Not applicable.

## References

[1] Annese T., Tamma R., Ruggieri S., Ribatti D. (2019). Erythropoietin in tumor angiogenesis. Exp. Cell Res..

[2] Tirpe A.A., Gulei D., Ciortea S.M., Crivii C., Berindan-Neagoe I. (2019). Hypoxia: Overview on hypoxia-mediated mechanisms with a focus on the role of HIF genes. Int. J. Mol. Sci..

[3] Yasuoka Y., Fukuyama T., Izumi Y., Nakayama Y., Inoue H., Yanagita K., Oshima T., Yamazaki T., Uematsu T., Kobayashi N. (2020). Erythropoietin production by the kidney and the liver in response to severe hypoxia evaluated by Western blotting with deglycosylation. Physiol. Rep..

[4] Kimáková P., Solár P., Solárová Z., Komel R., Debeljak N. (2017). Erythropoietin and its angiogenic activity. Int. J. Mol. Sci..

[5] Ostrowski D., Heinrich R. (2018). Alternative erythropoietin receptors in the nervous system. J. Clin. Med..

[6] Klopsch C., Skorska A., Ludwig M., Lemcke H., Maass G., Gaebel R., Beyer M., Lux C., Toelk A., Müller K. (2018). Intramyocardial angiogenetic stem cells and epicardial erythropoietin save the acute ischemic heart. Dis. Models Mech..

[7] Bretz C.A., Ramshekar A., Kunz E., Wang H., Hartnett M.E. (2020). Signaling through the erythropoietin receptor affects angiogenesis in retinovascular disease. Investig. Ophthalmol. Vis. Sci..

[8] Samson F.P., He W., Sripathi S.R., Patrick A.T., Madu J., Chung H., Frost M.C., Jee D., Gutsaeva D.R., Jahng W.J. (2020). Dual switch mechanism of erythropoietin as an antiapoptotic and pro-angiogenic determinant in the retina. ACS Omega.

[9] García-Ramírez M., Hernández C., Simó R. (2008). Expression of erythropoietin and its receptor in the human retina: A comparative study of diabetic and nondiabetic subjects. Diabetes Care.

[10] Dreixler J.C., Hagevik S., Hemmert J.W., Shaikh A.R., Rosenbaum D.M., Roth S. (2009). Involvement of erythropoietin in retinal ischemic preconditioning. Anesthesiology.

[11] Junk A.K., Mammis A., Savitz S.I., Singh M., Roth S., Malhotra S., Rosenbaum P.S., Cerami A., Brines M., Rosenbaum D.M. (2002). Erythropoietin administration protects retinal neurons from acute ischemia-reperfusion injury. Proc. Natl. Acad. Sci. USA.

[12] Chang Z.Y., Yeh M.K., Chiang C.H., Chen Y.H., Lu D.W. (2013). Erythropoietin protects adult retinal ganglion cells against NMDA-, trophic factor withdrawal-, and TNF-α-induced damage. PLoS ONE.

[13] Cheng W.S., Lin I.H., Feng K.M., Chang Z.Y., Huang Y.C., Lu D.W. (2020). Neuroprotective effects of exogenous erythropoietin in Wistar rats by downregulating apoptotic factors to attenuate N-methyl-D-aspartate-mediated retinal ganglion cells death. PLoS ONE.

[14] Si W., Wang J., Li M., Qu H., Gu R., Liu R., Wang L., Li S., Hu X. (2019). Erythropoietin protects neurons from apoptosis via activating PI3K/AKT and inhibiting Erk1/2 signaling pathway. 3 Biotech.

[15] Pathipati P., Ferriero D.M. (2017). The differential effects of erythropoietin exposure to oxidative stress on microglia and astrocytes in vitro. Dev. Neurosci..

[16] Zhou Z.W., Li F., Zheng Z.T., Li Y.D., Chen T.H., Gao W.W., Chen J.L., Zhang J.N. (2017). Erythropoietin regulates immune/inflammatory reaction and improves neurological function outcomes in traumatic brain injury. Brain Behav..

[17] Constantinescu S.N., Ghaffari S., Lodish H.F. (1999). The erythropoietin receptor: Structure, activation and intracellular signal transduction. Trends Endocrinol. Metab. TEM.

[18] Watowich S.S., Hilton D.J., Lodish H.F. (1994). Activation and inhibition of erythropoietin receptor function: Role of receptor dimerization. Mol. Cell. Biol..

[19] Kim A.R., Ulirsch J.C., Wilmes S., Unal E., Moraga I., Karakukcu M., Yuan D., Kazerounian S., Abdulhay N.J., King D.S. (2017). Functional selectivity in cytokine signaling revealed through a pathogenic EPO mutation. Cell.

[20] Tóthová Z., Tomc J., Debeljak N., Solár P. (2021). STAT5 as a key protein of erythropoietin signalization. Int. J. Mol. Sci..

[21] Tóthová Z., Šemeláková M., Solárová Z., Tomc J., Debeljak N., Solár P. (2021). The role of PI3K/AKT and MAPK signaling pathways in erythropoietin signalization. Int. J. Mol. Sci..

[22] Sanghera K.P., Mathalone N., Baigi R., Panov E., Wang D., Zhao X., Hsu H., Wang H., Tropepe V., Ward M. (2011). The PI3K/Akt/mTOR pathway mediates retinal progenitor cell survival under hypoxic and superoxide stress. Mol. Cell. Neurosci..

[23] Masuda S., Nagao M., Takahata K., Konishi Y., Gallyas F., Tabira T., Sasaki R. (1993). Functional erythropoietin receptor of the cells with neural characteristics. Comparison with receptor properties of erythroid cells. J. Biol. Chem..

[24] Kebschull L., Theilmann L.F.C., Mohr A., Uennigmann W., Stoeppeler S., Heitplatz B., Spiegel H.U., Bahde R., Palmes D.M., Becker F. (2017). EPOR_2_/βcR_2_-independendent effects of low-dose epoetin-α in porcine liver transplantation. Biosci. Rep..

[25] Jubinsky P.T., Krijanovski O.I., Nathan D.G., Tavernier J., Sieff C.A. (1997). The beta chain of the interleukin-3 receptor functionally associates with the erythropoietin receptor. Blood.

[26] Peng B., Kong G., Yang C., Ming Y. (2020). Erythropoietin and its derivatives: From tissue protection to immune regulation. Cell Death Dis..

[27] Colella P., Iodice C., Di Vicino U., Annunziata I., Surace E.M., Auricchio A. (2011). Non-erythropoietic erythropoietin derivatives protect from light-induced and genetic photoreceptor degeneration. Hum. Mol. Genet..

[28] Wang Z., Liu C.H., Huang S., Chen J. (2019). Wnt signaling in vascular eye diseases. Prog. Retin. Eye Res..

[29] Wagstaff P.E., Heredero Berzal A., Boon C.J.F., Quinn P.M.J., Ten Asbroek A., Bergen A.A. (2021). The role of small molecules and their effect on the molecular mechanisms of early retinal organoid development. Int. J. Mol. Sci..

[30] Vizcardo-Galindo G., León-Velarde F., Villafuerte F.C. (2020). High-altitude hypoxia decreases plasma erythropoietin soluble receptor concentration in lowlanders. High Alt. Med. Biol..

[31] Khankin E.V., Mutter W.P., Tamez H., Yuan H.T., Karumanchi S.A., Thadhani R. (2010). Soluble erythropoietin receptor contributes to erythropoietin resistance in end-stage renal disease. PLoS ONE.

[32] Zhang D., Lv F.L., Wang G.H. (2018). Effects of HIF-1α on diabetic retinopathy angiogenesis and VEGF expression. Eur. Rev. Med. Pharmacol. Sci..

[33] Ogawa C., Tsuchiya K., Tomosugi N., Maeda K. (2020). A hypoxia-inducible factor stabilizer improves hematopoiesis and iron metabolism early after administration to treat anemia in hemodialysis patients. Int. J. Mol. Sci..

[34] Socolovsky M., Nam H., Fleming M.D., Haase V.H., Brugnara C., Lodish H.F. (2001). Ineffective erythropoiesis in Stat5a(-/-)5b(-/-) mice due to decreased survival of early erythroblasts. Blood.

[35] Tu P.S., Lin E.C., Chen H.W., Chen S.W., Lin T.A., Gau J.P., Chang Y.I. (2020). The extracellular signal-regulated kinase 1/2 modulates the intracellular localization of DNA methyltransferase 3A to regulate erythrocytic differentiation. Am. J. Transl. Res..

[36] Dai T.Y., Lan J.J., Gao R.L., Zhao Y.N., Yu X.L., Liang S.X., Liu W.B., Sun X. (2022). Panaxdiol saponins component promotes hematopoiesis by regulating GATA transcription factors of intracellular signaling pathway in mouse bone marrow. Ann. Transl. Med..

[37] Das T.P., Suman S., Alatassi H., Ankem M.K., Damodaran C. (2016). Inhibition of AKT promotes FOXO3a-dependent apoptosis in prostate cancer. Cell Death Dis..

[38] Digicaylioglu M., Lipton S.A. (2001). Erythropoietin-mediated neuroprotection involves cross-talk between Jak2 and NF-kappaB signalling cascades. Nature.

[39] Shen J., Wu Y., Xu J.Y., Zhang J., Sinclair S.H., Yanoff M., Xu G., Li W., Xu G.T. (2010). ERK- and Akt-dependent neuroprotection by erythropoietin (EPO) against glyoxal-AGEs via modulation of Bcl-xL, Bax, and BAD. Investig. Ophthalmol. Vis. Sci..

[40] Chong Z.Z., Kang J.Q., Maiese K. (2003). Apaf-1, Bcl-xL, cytochrome c, and caspase-9 form the critical elements for cerebral vascular protection by erythropoietin. J. Cereb. Blood Flow Metab. Off. J. Int. Soc. Cereb. Blood Flow Metab..

[41] Wang Z.Y., Shen L.J., Tu L., Hu D.N., Liu G.Y., Zhou Z.L., Lin Y., Chen L.H., Qu J. (2009). Erythropoietin protects retinal pigment epithelial cells from oxidative damage. Free Radic. Biol. Med..

[42] Wang C.Y., Mayo M.W., Korneluk R.G., Goeddel D.V., Baldwin A.S. (1998). NF-kappaB antiapoptosis: Induction of TRAF1 and TRAF2 and c-IAP1 and c-IAP2 to suppress caspase-8 activation. Science.

[43] Chen C., Edelstein L.C., Gélinas C. (2000). The Rel/NF-kappaB family directly activates expression of the apoptosis inhibitor Bcl-x(L). Mol. Cell. Biol..

[44] Chen S., Guttridge D.C., You Z., Zhang Z., Fribley A., Mayo M.W., Kitajewski J., Wang C.Y. (2001). Wnt-1 signaling inhibits apoptosis by activating beta-catenin/T cell factor-mediated transcription. J. Cell Biol..

[45] Duda P., Akula S.M., Abrams S.L., Steelman L.S., Martelli A.M., Cocco L., Ratti S., Candido S., Libra M., Montalto G. (2020). Targeting GSK3 and associated signaling pathways involved in cancer. Cells.

[46] Kwak J., Kim J.H., Jang H.N., Jung M.H., Cho H.S., Chang S.H., Kim H.J. (2020). Erythropoietin ameliorates ischemia/reperfusion-induced acute kidney injury via inflammasome suppression in mice. Int. J. Mol. Sci..

[47] Gong Q., Zeng J., Zhang X., Huang Y., Chen C., Quan J., Ling J. (2021). Effect of erythropoietin on angiogenic potential of dental pulp cells. Exp. Ther. Med..

[48] Lin X., Ma X., Cui X., Zhang R., Pan H., Gao W. (2020). Effects of erythropoietin on lung injury induced by cardiopulmonary bypass after cardiac surgery. Med. Sci. Monit. Int. Med. J. Exp. Clin. Res..

[49] Cui J., Zhang F., Cao W., Wang Y., Liu J., Liu X., Chen T., Li L., Tian J., Yu B. (2018). Erythropoietin alleviates hyperglycaemia-associated inflammation by regulating macrophage polarization via the JAK2/STAT3 signalling pathway. Mol. Immunol..

[50] Elshiekh M., Kadkhodaee M., Seifi B., Ranjbaran M., Askari H. (2017). Up-regulation of nitric oxide synthases by erythropoietin alone or in conjunction with ischemic preconditioning in ischemia reperfusion injury of rat kidneys. Gen. Physiol. Biophys..

[51] Cruz Navarro J., Pillai S., Ponce L.L., Van M., Goodman J.C., Robertson C.S. (2014). Endothelial nitric oxide synthase mediates the cerebrovascular effects of erythropoietin in traumatic brain injury. Front. Immunol..

[52] Govindappa P.K., Elfar J.C. (2022). Erythropoietin promotes M2 macrophage phagocytosis of Schwann cells in peripheral nerve injury. Cell Death Dis..

[53] Einwächter H., Heiseke A., Schlitzer A., Gasteiger G., Adler H., Voehringer D., Manz M.G., Ruzsics Z., Dölken L., Koszinowski U.H. (2020). The innate immune response to infection induces erythropoietin-dependent replenishment of the dendritic cell compartment. Front. Immunol..

[54] Korkmaz T., Kahramansoy N., Kilicgun A., Firat T. (2014). The effect of erythropoietin to pulmonary injury and mast cells secondary to acute pancreatitis. BMC Res. Notes.

[55] Cantarelli C., Angeletti A., Cravedi P. (2019). Erythropoietin, a multifaceted protein with innate and adaptive immune modulatory activity. Am. J. Transpl. Off. J. Am. Soc. Transpl. Am. Soc. Transpl. Sur..

[56] Purroy C., Fairchild R.L., Tanaka T., Baldwin W.M., Manrique J., Madsen J.C., Colvin R.B., Alessandrini A., Blazar B.R., Fribourg M. (2017). Erythropoietin receptor-mediated molecular crosstalk promotes T cell immunoregulation and transplant survival. J. Am. Soc. Nephrol. JASN.

[57] Arıkan T., Akcan A., Dönder Y., Yılmaz Z., Sözüer E., Öz B., Baykan M., Gök M., Poyrazoğlu B. (2019). Effects of erythropoietin on bacterial translocation in a rat model of experimental colitis. Turk. J. Surg..

[58] Moransard M., Bednar M., Frei K., Gassmann M., Ogunshola O.O. (2017). Erythropoietin reduces experimental autoimmune encephalomyelitis severity via neuroprotective mechanisms. J. Neuroinflamm..

[59] Shokrzadeh M., Etebari M., Ghassemi-Barghi N. (2020). An engineered non-erythropoietic erythropoietin-derived peptide, ARA290, attenuates doxorubicin induced genotoxicity and oxidative stress. Toxicol. Vitr. Int. J. Publ. Assoc. BIBRA.

[60] Dang J.Z., Tu Y.F., Wang J., Yang Y.J. (2021). Carbamylated erythropoietin alleviates kidney damage in diabetic rats by suppressing oxidative stress. Curr. Med. Sci..

[61] Salinas M., Wang J., Rosa de Sagarra M., Martín D., Rojo A.I., Martin-Perez J., Ortiz de Montellano P.R., Cuadrado A. (2004). Protein kinase Akt/PKB phosphorylates heme oxygenase-1 in vitro and in vivo. FEBS Lett..

[62] Diaz Z., Assaraf M.I., Miller W.H., Schipper H.M. (2005). Astroglial cytoprotection by erythropoietin pre-conditioning: Implications for ischemic and degenerative CNS disorders. J. Neurochem..

[63] Thompson A.M., Farmer K., Rowe E.M., Hayley S. (2020). Erythropoietin modulates striatal antioxidant signalling to reduce neurodegeneration in a toxicant model of Parkinson’s disease. Mol. Cell. Neurosci..

[64] Glass G.A., Gershon D. (1984). Decreased enzymic protection and increased sensitivity to oxidative damage in erythrocytes as a function of cell and donor aging. Biochem. J..

[65] Bany-Mohammed F.M., Slivka S., Hallman M. (1996). Recombinant human erythropoietin: Possible role as an antioxidant in premature rabbits. Pediatr. Res..

[66] Pulukool S.K., Bhagavatham S.K.S., Kannan V., Sukumar P., Dandamudi R.B., Ghaisas S., Kunchala H., Saieesh D., Naik A.A., Pargaonkar A. (2021). Elevated dimethylarginine, ATP, cytokines, metabolic remodeling involving tryptophan metabolism and potential microglial inflammation characterize primary open angle glaucoma. Sci. Rep..

[67] Krishnan A., Kocab A.J., Zacks D.N., Marshak-Rothstein A., Gregory-Ksander M. (2019). A small peptide antagonist of the Fas receptor inhibits neuroinflammation and prevents axon degeneration and retinal ganglion cell death in an inducible mouse model of glaucoma. J. Neuroinflamm..

[68] Li Q., Jin R., Zhang S., Sun X., Wu J. (2020). Group II metabotropic glutamate receptor agonist promotes retinal ganglion cell survival by reducing neuronal excitotoxicity in a rat chronic ocular hypertension model. Neuropharmacology.

[69] Cha Y.W., Kim S.T. (2021). Serum and aqueous humor levels of brain-derived neurotrophic factor in patients with primary open-angle glaucoma and normal-tension glaucoma. Int. Ophthalmol..

[70] Mokbel T.H., Ghanem A.A., Kishk H., Arafa L.F., El-Baiomy A.A. (2010). Erythropoietin and soluble CD44 levels in patients with primary open-angle glaucoma. Clin. Exp. Ophthalmol..

[71] Arjamaa O., Nikinmaa M. (2006). Oxygen-dependent diseases in the retina: Role of hypoxia-inducible factors. Exp. Eye Res..

[72] Kawakami M., Sekiguchi M., Sato K., Kozaki S., Takahashi M. (2001). Erythropoietin receptor-mediated inhibition of exocytotic glutamate release confers neuroprotection during chemical ischemia. J. Biol. Chem..

[73] Chader G.J. (2012). Advances in glaucoma treatment and management: Neurotrophic agents. Investig. Opthalmol. Vis. Sci..

[74] Cheng S., Wang H.N., Xu L.J., Li F., Miao Y., Lei B., Sun X., Wang Z. (2021). Soluble tumor necrosis factor-alpha-induced hyperexcitability contributes to retinal ganglion cell apoptosis by enhancing Nav1.6 in experimental glaucoma. J. Neuroinflamm..

[75] Zhong L., Bradley J., Schubert W., Ahmed E., Adamis A.P., Shima D.T., Robinson G.S., Ng Y.S. (2007). Erythropoietin promotes survival of retinal ganglion cells in DBA/2J glaucoma mice. Investig. Opthalmol. Vis. Sci..

[76] Resende A.P., Rosolen S.G., Nunes T., São Braz B., Delgado E. (2018). Functional and structural effects of erythropoietin subconjunctival administration in glaucomatous animals. Biomed. Hub.

[77] Zhong Y.S., Liu X.H., Cheng Y., Min Y.J. (2008). Erythropoietin with retrobulbar administration protects retinal ganglion cells from acute elevated intraocular pressure in rats. J. Ocul. Pharmacol. Ther. Off. J. Assoc. Ocul. Pharmacol. Ther..

[78] Tsai J.C., Wu L., Worgul B., Forbes M., Cao J. (2005). Intravitreal administration of erythropoietin and preservation of retinal ganglion cells in an experimental rat model of glaucoma. Curr. Eye Res..

[79] Zhou M., Chen S., Wang W., Huang W., Cheng B., Ding X., Zhang X. (2013). Levels of erythropoietin and vascular endothelial growth factor in surgery-required advanced neovascular glaucoma eyes before and after intravitreal injection of bevacizumab. Investig. Opthalmol. Vis. Sci..

[80] Sun Y., Zhao H., Shen Y., Guan W. (2019). Comparison of erythropoietin, semaphorins 3A and pigment epithelium derived factor levels in serum and aqueous humor of patients with neovascular glaucoma and cataract. J. Coll. Phys. Surg. Pak. JCPSP.

[81] Watanabe D., Suzuma K., Matsui S., Kurimoto M., Kiryu J., Kita M., Suzuma I., Ohashi H., Ojima T., Murakami T. (2005). Erythropoietin as a retinal angiogenic factor in proliferative diabetic retinopathy. N. Engl. J. Med..

[82] Mackay D.D. (2015). Should patients with optic neuritis be treated with steroids?. Curr. Opin. Ophthalmol..

[83] Diem R., Hobom M., Maier K., Weissert R., Storch M.K., Meyer R., Bähr M. (2003). Methylprednisolone increases neuronal apoptosis during autoimmune CNS inflammation by inhibition of an endogenous neuroprotective pathway. J. Neurosci. Off. J. Soc. Neurosci..

[84] Sättler M.B., Merkler D., Maier K., Stadelmann C., Ehrenreich H., Bähr M., Diem R. (2004). Neuroprotective effects and intracellular signaling pathways of erythropoietin in a rat model of multiple sclerosis. Cell Death Differ..

[85] Soltan Sanjari M., Pakdel F., Moosavi F., Pirmarzdashti N., Nojomi M., Haghighi A., Hashemi M., Bahmani Kashkouli M. (2019). Visual outcomes of adding erythropoietin to methylprednisolone for treatment of retrobulbar optic neuritis. J. Ophthalmic Vis. Res..

[86] Shayegannejad V., Shahzamani S., Dehghani A., Dast Borhan Z., Rahimi M., Mirmohammadsadeghi A. (2015). A double-blind, placebo-controlled trial of adding erythropoietin to intravenous methylprednisolone for the treatment of unilateral acute optic neuritis of unknown or demyelinative origin. Graefe’s Archive Clin. Exp. Ophthalmol..

[87] Sühs K.W., Hein K., Sättler M.B., Görlitz A., Ciupka C., Scholz K., Käsmann-Kellner B., Papanagiotou P., Schäffler N., Restemeyer C. (2012). A randomized, double-blind, phase 2 study of erythropoietin in optic neuritis. Ann. Neurol..

[88] Lagrèze W.A., Küchlin S., Ihorst G., Grotejohann B., Beisse F., Volkmann M., Heinrich S.P., Albrecht P., Ungewiss J., Wörner M. (2021). Safety and efficacy of erythropoietin for the treatment of patients with optic neuritis (TONE): A randomised, double-blind, multicentre, placebo-controlled study. Lancet Neurol..

[89] Modarres M., Falavarjani K.G., Nazari H., Sanjari M.S., Aghamohammadi F., Homaii M., Samiy N. (2011). Intravitreal erythropoietin injection for the treatment of non-arteritic anterior ischaemic optic neuropathy. Br. J. Ophthalmol..

[90] Pakravan M., Esfandiari H., Hassanpour K., Razavi S., Pakravan P. (2017). The effect of combined systemic erythropoietin and steroid on non-arteritic anterior ischemic optic neuropathy: A prospective study. Curr. Eye Res..

[91] Nikkhah H., Golalipour M., Doozandeh A., Pakravan M., Yaseri M., Esfandiari H. (2020). The effect of systemic erythropoietin and oral prednisolone on recent-onset non-arteritic anterior ischemic optic neuropathy: A randomized clinical trial. Graefe’s Archive Clin. Exp. Ophthalmol..

[92] Kashkouli M.B., Pakdel F., Sanjari M.S., Haghighi A., Nojomi M., Homaee M.H., Heirati A. (2011). Erythropoietin: A novel treatment for traumatic optic neuropathy-a pilot study. Graefe’s Archive Clin. Exp. Ophthalmol..

[93] Entezari M., Esmaeili M., Yaseri M. (2014). A pilot study of the effect of intravenous erythropoetin on improvement of visual function in patients with recent indirect traumatic optic neuropathy. Graefe’s Archive Clin. Exp. Ophthalmol..

[94] Kashkouli M.B., Yousefi S., Nojomi M., Sanjari M.S., Pakdel F., Entezari M., Etezad-Razavi M., Razeghinejad M.R., Esmaeli M., Shafiee M. (2018). Traumatic optic neuropathy treatment trial (TONTT): Open label, phase 3, multicenter, semi-experimental trial. Graefe’s Archive Clin. Exp. Ophthalmol..

[95] Rashad M.A., Abdel Latif A.A.M., Mostafa H.A., Fawzy S.M., Abdel Latif M.A.M. (2018). Visual-evoked-response-supported outcome of intravitreal erythropoietin in management of indirect traumatic optic neuropathy. J. Ophthalmol..

[96] Wladis E.J., Aakalu V.K., Sobel R.K., McCulley T.J., Foster J.A., Tao J.P., Freitag S.K., Yen M.T. (2021). Interventions for indirect traumatic optic neuropathy: A report by the American academy of ophthalmology. Ophthalmology.

[97] Anand S., Al-Mondhiry J., Fischer K., Glaspy J. (2021). Epoetin alfa-epbx: A new entrant into a crowded market. a historical review of the role of erythropoietin stimulating agents and the development of the first epoetin biosimilar in the United States. Expert Rev. Clin. Pharmacol..

[98] Lee D.E., Son W., Ha B.J., Oh M.S., Yoo O.J. (2006). The prolonged half-lives of new erythropoietin derivatives via peptide addition. Biochem. Biophys. Res. Commun..

[99] Powell J., Gurk-Turner C. (2002). Darbepoetin alfa (Aranesp). Bayl. Univ. Med. Cent. Proc..

[100] Aizawa K., Kawasaki R., Tashiro Y., Hirata M., Endo K., Shimonaka Y. (2016). Epoetin beta pegol, but not recombinant erythropoietin, retains its hematopoietic effect in vivo in the presence of the sialic acid-metabolizing enzyme sialidase. Int. J. Hematol..

[101] Liu X., Zhu B., Zou H., Hu D., Gu Q., Liu K., Xu X. (2015). Carbamylated erythropoietin mediates retinal neuroprotection in streptozotocin-induced early-stage diabetic rats. Graefe’s Archive Clin. Exp. Ophthalmol..

[102] Chen J., Yang Z., Zhang X. (2015). Carbamylated erythropoietin: A prospective drug candidate for neuroprotection. Biochem. Insights.

[103] McVicar C.M., Hamilton R., Colhoun L.M., Gardiner T.A., Brines M., Cerami A., Stitt A.W. (2011). Intervention with an erythropoietin-derived peptide protects against neuroglial and vascular degeneration during diabetic retinopathy. Diabetes.

[104] Zhang C., Yang C., Zhu T. (2017). From erythropoietin to its peptide derivatives: Smaller but stronger. Curr. Protein Pept. Sci..

[105] Cho B., Yoo S.J., Kim S.Y., Lee C.H., Lee Y.I., Lee S.R., Moon C. (2022). Second-generation non-hematopoietic erythropoietin-derived peptide for neuroprotection. Redox Biol..

[106] Silva B., Marto J., Braz B.S., Delgado E., Almeida A.J., Gonçalves L. (2020). New nanoparticles for topical ocular delivery of erythropoietin. Int. J. Pharm..

[107] DeJulius C.R., Bernardo-Colón A., Naguib S., Backstrom J.R., Kavanaugh T., Gupta M.K., Duvall C.L., Rex T.S. (2021). Microsphere antioxidant and sustained erythropoietin-R76E release functions cooperate to reduce traumatic optic neuropathy. J. Control. Release Off. J. Control. Release Soc..

[108] Bond W.S., Hines-Beard J., GoldenMerry Y.L., Davis M., Farooque A., Sappington R.M., Calkins D.J., Rex T.S. (2016). Virus-mediated EpoR76E therapy slows optic nerve axonopathy in experimental glaucoma. Mol. Ther. J. Am. Soc. Gene Ther..

[109] Tao Y., Zhu Q., Wang L., Zha X., Teng D., Xu L. (2020). Adeno-associated virus (AAV)-mediated neuroprotective effects on the degenerative retina: The therapeutic potential of erythropoietin. Fundam. Clin. Pharmacol..

[110] Hines-Beard J., Desai S., Haag R., Esumi N., D’Surney L., Parker S., Richardson C., Rex T.S. (2013). Identification of a therapeutic dose of continuously delivered erythropoietin in the eye using an inducible promoter system. Curr. Gene Ther..

[111] Zhong G., Wang H., He W., Li Y., Mou H., Tickner Z.J., Tran M.H., Ou T., Yin Y., Diao H. (2020). A reversible RNA on-switch that controls gene expression of AAV-delivered therapeutics in vivo. Nat. Biotechnol..

[112] Bohr S., Patel S.J., Vasko R., Shen K., Iracheta-Vellve A., Lee J., Bale S.S., Chakraborty N., Brines M., Cerami A. (2015). Modulation of cellular stress response via the erythropoietin/CD131 heteroreceptor complex in mouse mesenchymal-derived cells. J. Mol. Med..

[113] He L., Cohen E.B., Edwards A.P.B., Xavier-Ferrucio J., Bugge K., Federman R.S., Absher D., Myers R.M., Kragelund B.B., Krause D.S. (2019). Transmembrane protein aptamer induces cooperative signaling by the EPO receptor and the cytokine receptor β-common subunit. iScience.

[114] Liu W., Rong Y., Wang J., Zhou Z., Ge X., Ji C., Jiang D., Gong F., Li L., Chen J. (2020). Exosome-shuttled miR-216a-5p from hypoxic preconditioned mesenchymal stem cells repair traumatic spinal cord injury by shifting microglial M1/M2 polarization. J. Neuroinflamm..

[115] Kim J., Lee Y., Lee S., Kim K., Song M., Lee J. (2020). Mesenchymal stem cell therapy and alzheimer’s disease: Current status and future perspectives. J. Alzheimer’s Dis. JAD.

[116] Luque-Campos N., Contreras-López R.A., Jose Paredes-Martínez M., Torres M.J., Bahraoui S., Wei M., Espinoza F., Djouad F., Elizondo-Vega R.J., Luz-Crawford P. (2019). Mesenchymal stem cells improve rheumatoid arthritis progression by controlling memory T cell response. Front. Immunol..

[117] Jiang Y., Gao H., Yuan H., Xu H., Tian M., Du G., Xie W. (2019). Amelioration of postoperative cognitive dysfunction in mice by mesenchymal stem cell-conditioned medium treatments is associated with reduced inflammation, oxidative stress and increased BDNF expression in brain tissues. Neurosci. Lett..

[118] Hu Y., Liang J., Cui H., Wang X., Rong H., Shao B., Cui H. (2013). Wharton’s jelly mesenchymal stem cells differentiate into retinal progenitor cells. Neural Regen. Res..

[119] Johnson T.V., Bull N.D., Hunt D.P., Marina N., Tomarev S.I., Martin K.R. (2010). Neuroprotective effects of intravitreal mesenchymal stem cell transplantation in experimental glaucoma. Investig. Opthalmol. Vis. Sci..

[120] Daga A., Muraglia A., Quarto R., Cancedda R., Corte G. (2002). Enhanced engraftment of EPO-transduced human bone marrow stromal cells transplanted in a 3D matrix in non-conditioned NOD/SCID mice. Gene Ther..

[121] Omoto M., Katikireddy K.R., Rezazadeh A., Dohlman T.H., Chauhan S.K. (2014). Mesenchymal stem cells home to inflamed ocular surface and suppress allosensitization in corneal transplantation. Investig. Opthalmol. Vis. Sci..

[122] Shirley Ding S.L., Kumar S., Ali Khan M.S., Ling Mok P. (2018). Human mesenchymal stem cells expressing erythropoietin enhance survivability of retinal neurons against oxidative stress: An in vitro study. Front. Cell. Neurosci..

